# 

**Published:** 1901-10

**Authors:** 


### BOOKS RECEIVED.

A Handbook of Pathological Anatomy and Histology. With an Introductory Section on Post Morten Examinations and the Methods of Preserving and Examining Diseased Tissues. By Francis Delafield, M. D., LL. D., Professor of the Practice of Medicine, College of Physicians and Surgeons, Columbia University, New York. And T. Mitchell Prudden, M D , LL. D , Professor of Pathology, College of Physicians and Surgeons, New York. Sixth edition. With 13 full-page plates and 453 illustrations in the text in black and colors. Royal 8vo, pp. 839 New York: William Wood & Company. 1901. [Price, muslin, $5.00 net; sheep, $5.75 net.]

A Text-Book of the Practice of Medicine. By James M. Anders, M. D., Ph.D., LL. D., Professor of the Practice of Medicine and of Clinical Medicine, Medico-Chirurgical College, Philadelphia. Fifth edition, thoroughly revised. One handsome 8vo volume of 1,297 pages, fully illustrated. Philadelphia and London: W. B. Saunders & Co. 1901. [Price, cloth, $5.50 net.]

The Mental Functions of the Brain. An Investigation into their Localisation and their Manifestation in Health and Disease. By Bernard Hollander, M. D., M. R. C S., L. R. C. P. (London). Illustrated with clinical records of 800 cases of localised brain derangements and with several plates. Octavo, pp. 523. New York and London: G. P. Putnam's Sons. 1901.

A Guide to the Clinical Examination of the Blood for Diagnostic Purposes. By Richard C Cabot, M. D. With colored plates and engravings. Fourth revised edition. Octavo, pp. 515. New York: William Wood & Company. 1901.

Twenty-seventh Annual Report of the State Board of Health of the State of Michigan for the Fiscal Year Ending June 30, 1899. Henry B. Baker, M. D., Secretary. 1901.

Mt. Sinai Hospital Reports. Volume II. For 1899 and 1900. Edited by Paul F. Mundé, M. D. New York. 1901.

Transactions of the Medical Society of the State of New York at its Ninety-fifth Annual Session, held at Albany, January 29, 30 and 31, 1901. Frederic C. Curtis, M. D., Secretary. Published by the Society. 1901.

The Pathology and Treatment of Sexual Impotence. By Victor G. Vecki, M. D. Third edition, revised and enlarged Duodecimo, 329 pages. Philadelphia and London: W. B. Saunders & Company. 1901. [Price, cloth, $2.00 net.]

Nervous and Mental Diseases. By Archibald Church, M. D., Professor of Nervous and Mental Diseases and Head of Neurological Department, Northwestern University Medical School; and Frederick Peterson, M. D., Chief of Clinic, Department of Nervous and Mental Diseases, and Clinical Lecturer on Psychiatry, College of Physicians and Surgeons, New York. Third edition, revised and enlarged. Handsome 8vo volume of 870 pages, with 322 illustrations. Philadelphia and London: W. B. Saunders & Company. 1901. [Price, cloth, $5.00 net.]

The American Illustrated Medical Dictionary. For Practitioners and Students. A Complete Dictionary of the Terms used in Medicine, Surgery, Dentistry, Pharmacy, Chemistry, and the kindred branches, including much collateral information of an encyclopedic character, together with new and elaborate tables of Arteries, Muscles, Nerves, Veins, etc.; of Bacilli, Bacteria, Micrococci, Streptococci; Eponymic Tables of Diseases, Operations, Signs and Symptoms, Stains, Tests, Methods of Treatment, etc., etc. By W. A. Newman Dorland, A. M., M. D, editor of the "American Pocket Medical Dictionary." Second edition, revised. Handsome large 8vo, nearly 800 pages, bound in full flexible leather. Philadelphia and London: W. B. Saunders & Company. 1901 [Price, $4.50 net.]

Manual of the Diseases of the Eye. For Students and General Practitioners. By Charles H. May, M. D., Chief of Clinic and Instructor in Ophthalmology, Eye Department, College of Physicians and Surgeons, New York. Second edition, revised. 12mo, pp. 421. 275 original illustrations, including 36 colored figures. New York: William Wood & Company. 1901.

